# Supplementary material for: CDK4/6 inhibitor-induced liver injury: Clinical phenotypes and role of corticosteroid treatment
Source: JHEP Rep. 2024 Apr 16;6(7):101098. doi: 10.1016/j.jhepr.2024.101098 (PMC11220524; doi:10.1016/j.jhepr.2024.101098)
Supplement: Multimedia component 1 [file mmc1.pdf]

CDK4/6 inhibitor-induced liver injury: Clinical phenotypes and role of corticosteroid treatment

Lucy Meunier, Eleonora De Martin, Bénédicte Delire, Wiliam Jacot, Severine Guiu, Amel Zahhaf, Dominique Larrey, Yves Horsmans

Table of contents

Fig. S1……………………………………………………………………………………2

Fig. S2……………………………………………………………………………………2

Fig. S1. Onset of hepatitis after CDK4/6 inhibitors

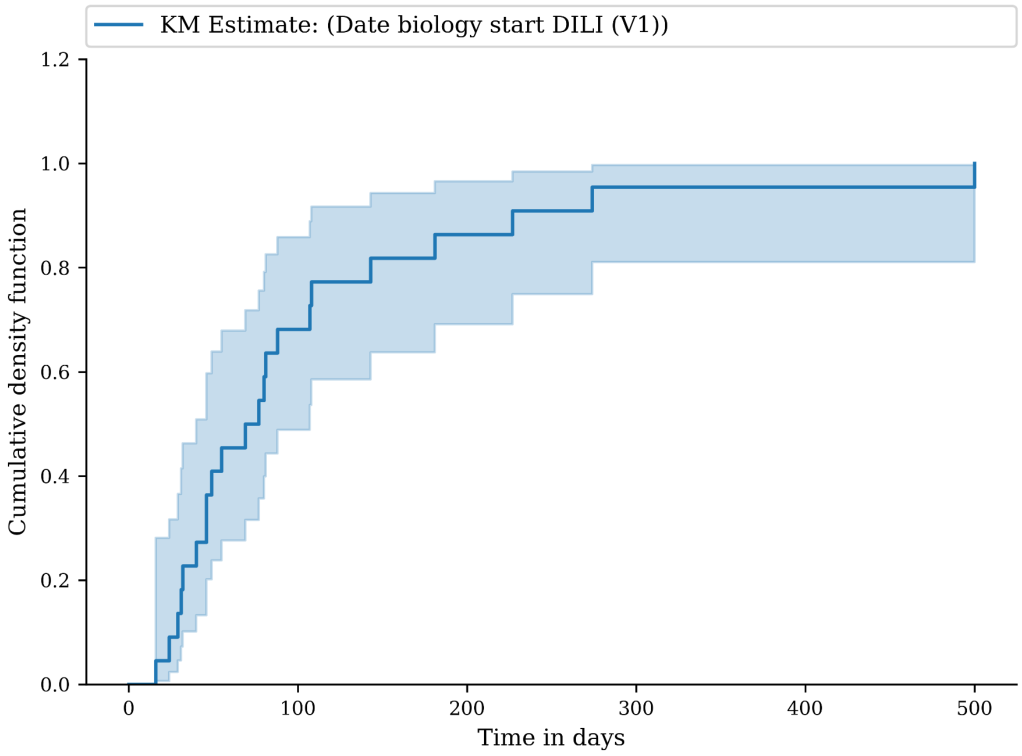

Fig. S2. Improvement of hepatitis after stopping CDK4/6 inhibitors

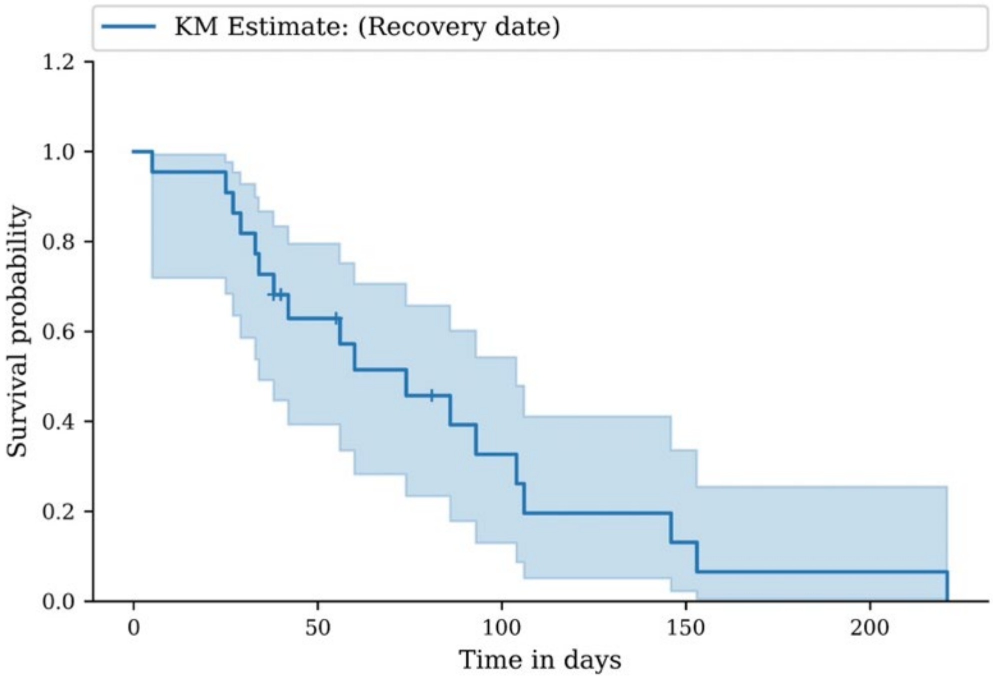

KM Estimate:

At risk	22	12	5	2	1
Censored	0	2	4	4	4
Events	0	8	13	16	17
