# Supplementary material for: CDK4/6 inhibitor-induced liver injury: Clinical phenotypes and role of corticosteroid treatment
Source: JHEP Rep. 2024 Apr 16;6(7):101098. doi: 10.1016/j.jhepr.2024.101098 (PMC11220524; doi:10.1016/j.jhepr.2024.101098)
Supplement: Multimedia component 4 [file mmc4.pdf]

CDK4/6 inhibitor-induced liver injury: Clinical phenotypes and role of corticosteroid treatment

Lucy Meunier[1,*], Eleonora De Martin[2], Bénédicte Delire[3], Wiliam Jacot[4], Severine Guiu[4], Amel Zahhaf[1], Dominique Larrey[1], Yves Horsmans[3]

JHEP Reports **2024**. vol. 6 | 1–7

Check for updates

Background & Aims: Cyclin-dependent kinase 4/6 (CDK4/6) inhibitors are the cornerstone of systemic therapy for patients with hormone receptor-positive, HER2-negative (HR+/HER2-) metastatic breast cancer. In the various therapeutic studies with CDK4/6 inhibitors, elevations in liver tests were more frequent than in the control groups. The mechanism of CDK4/6 inhibitor-induced liver toxicity is not well understood; moreover, natural history and appropriate management are poorly described.

Methods: We conducted a retrospective study, collecting cases of CDK4/6 hepatitis from the REFHEPS (Réseau Francophone pour l'étude de l'HEpatotoxicité des Produits de Santé) database.

Results: In this study, we report on 22 cases of hepatitis induced by CDK4/6 inhibitors (ribociclib, n = 19 and abemaciclib, n = 3). According to the CTCAE classification, all hepatitis cases were grade 3 or 4. Twelve (54.6%) patients had a liver biopsy showing acute centrilobular hepatitis with foci of necrosis and lymphocytic infiltrate. Nine (40.9%) patients were treated with corticosteroids for resolution of hepatitis. In three cases, another CDK4/6 inhibitor could be resumed after resolution of the hepatitis without recurrence.

Conclusions: CDK4/6 inhibitor-induced hepatitis is poorly described in the literature but there are several arguments pointing out that these drugs should be included in the DI-ALH (drug-induced autoimmune-like hepatitis) category.

© 2024 The Authors. Published by Elsevier B.V. on behalf of European Association for the Study of the Liver (EASL). This is an open access article under the CC BY license (http://creativecommons.org/licenses/by/4.0/).

Introduction

Cyclin-dependent kinase 4/6 (CDK4/6) inhibitors and endocrine therapy are the cornerstone of systemic therapy for patients with hormone receptor-positive (HR+) and human epidermal growth factor receptor-2-negative (HER2-) metastatic breast cancer. The Cyclin D-CDK4/6 protein complex effectively inhibits the Rb protein through phosphorylation allowing the progression of the cellular cycle from G1 to S phase. The inhibition of CDK4/6 activity stops cell division, thus CDK4/6 inhibitors prevent cancer cells from replicating. To date, three such molecules have been approved in the metastatic setting: palbociclib, ribociclib, and abemaciclib. The most common adverse effects are neutropenia for palbociclib and ribociclib and gastrointestinal toxicity for abemaciclib. In the various therapeutic studies with CDK4/6 inhibitors, elevations in liver tests were more frequent than in the control groups. For example, in the MONALEESA-2, MONALEESA-3 and MONALEESA-7 trials, 5 to 9.4% of patients treated with ribociclib experienced a grade 3 or 4 increase in serum alanine aminotransferase (ALT), according to CTCAE version 4.03, compared to 0.4 to 1.2% of patients in the control arm.[1] In the MONARCH-2 and MONARCH-3 trials, grade 3/4 ALT increase also occurred in more patients treated with abemaciclib (4.1-6.4%) compared to the control group (1.8-1.9%).[2,3] However, data from the PALOMA-2 and PALOMA-3 studies suggested that palbociclib was associated with slightly less frequent and less severe ALT elevations.[4,5] No cases of CDK4/6 inhibitor-related acute liver injury or liver-related death have been reported in these trials. In these studies, in the case of increased transaminases, CDK4/6 inhibitors were discontinued and resumed after liver test decreases (at reduced doses).

CDK4/6 inhibitors share different chemical and pharmacokinetic characteristics, including metabolism mediated by CYP3A4 (with the production of intermediate active metabolites, potentially leading to drug-drug interactions), and biliary clearance as the main elimination pathway. The mechanism of CDK4/6 inhibitor-induced liver toxicity is currently not known; moreover, natural history and appropriate management are poorly described.

The aim of the current study was to describe the clinical and biochemical features of CDK4/6-induced liver injury, its course and management.

Materials and methods

Study population

The Réseau Francophone pour l'étude de l'HEpatotoxicité des Produits de Santé (REFHEPS) is a French-speaking network for the study of the hepatotoxicity of health products created in January 2022. Each case is adjudicated by the REFHEPS

* Corresponding author. Address: 80 avenue Augmentin Fliche, 34080 Montpellier, France; Tel.: 04 67 33 02 24.
E-mail address: Lucy.meunier@chu-montpellier.fr (L. Meunier).
https://doi.org/10.1016/j.jhepr.2024.101098

committee, composed of five French and Belgian hepatologists with expertise in drug-induced liver injury (DILI).

The network uses a platform that allows all registered physicians to report a case of hepatotoxicity or request a diagnostic opinion or help with management.

All patients assessed by the REFHEPS network between January 1st 2022 and August 31st 2023 were evaluated and patients with CDK4/6 inhibitor-related liver toxicity were included in this study.

Hepatotoxicity definition

Liver injury was defined as serum ALT or aspartate aminotransferase (AST) ≥5x the upper limit of normal (ULN) and/or serum alkaline phosphatase (ALP) ≥2x the ULN or total bilirubin ≥2x the ULN associated with ALTx3 ULN according to the baseline value at initiation of the drug.[6] Type of liver injury was categorized by R ratios, using initial values of ALT divided by ALP, both expressed as ratios to the ULN: ALT/ULN ÷ ALP/ULN. R ratio >5 was categorized as hepatocellular, 2-5 as mixed and <2 as cholestatic.[6,7] Recovery was defined by complete normalization of liver tests.

Causality assessment of liver injury

A complete etiological work-up was performed in all patients including: viral hepatitis serologies (HAV, HBV, HCV, HEV), EBV, CMV, VZV, HSV, auto-antibodies, IgG level, liver imaging. Liver biopsy was performed at the discretion of the referring physician. Other specific causes of liver enzyme elevations were excluded.

The Roussel-Uclaf causality assessment (RUCAM) method[8] was used to assess causality, and the DILI network[9] score and CTCAE v5[10] were used to assess the severity of liver injury.

Statistical analysis

Descriptive statistics are presented as medians (ranges) for quantitative variables and counts (percentages) for qualitative variables. The Wilcoxon rank sum test was applied to compare the distribution of continuous variables and Chi-squared test (or Fisher's exact test when appropriate) was used to test the association of categorical variables. A p value <0.05 was considered statistically significant and all statistical tests were two-sided. We used the Kaplan-Meier method to estimate survival probabilities from 'date of biological onset of DILI (V1)' to 'date of recovery' and their point-wise 95% CIs.

Statistical analysis was performed using EasyMedStat (version 3.28). This study was approved by an ethic committee, IRB number: 198711.

Results

Between January 1st 2022 and August 31st 2023, 192 suspected DILI cases were submitted to and discussed within the REFHEPS network. Among them, 22 patients were treated with CDK4/6 inhibitors: 20 patients collected prospectively, and two patients collected retrospectively after reporting to REFHEPS. They were all women of median age 60 years (IQR 47.5–73.25) being treated for metastatic (n = 21) or locally advanced (n = 1) HR+/HER2-breast cancer (Table 1). Five patients had liver metastases. Ribociclib was involved in 19 cases and abemaciclib in three. Eighteen patients were treated with ribociclib 600 mg daily, whereas one patient was treated with 200 mg daily due to renal impairment. The three patients treated with abemaciclib received 300 mg daily (optimal dose). No

Table 1. Characteristics of all patients with anti-CDK4/6-induced liver injury.

Characteristics	Anti-CDK4/6-induced liver injury, n = 22
Age, years, mean (±SD) (min-max)	60.2 (13.8) (41-83)
Gender, n (%)	
Female	22 (100)
CDK4/6 inhibitor, n (%)	
Ribociclib	19 (86.4)
Abemaciclib	3 (13.6)
Liver metastasis, n (%)	5 (22.7)
Hormonal treatment, n (%)	
Letrozole	6 (27.3)
Letrozole/decapeptyl	4 (18.2)
Letrozole/enantone	3 (13.6)
Letrozole/gosereline	1 (4.5)
Anastrozole	2 (9.1)
Examestane	1 (4.5)
Fulvestrant	4 (18.2)
Time anti-CDK4/6 - DILI (days), mean (SD) (min-max)	104.7 (110.9) (55.5-153.8)
Clinical presentation, n (%)	
Asymptomatic	18 (81.8)
Nausea/asthenia	3 (13.6)
Pruritus	1 (4.5)
Autoimmune features, n (%)	
No	15 (68.2)
ANA ≥1/80	5 (22.7)
AAN 1/160 - Anti-mitochondrial antibodies 1/320 - IgG > N	1 (4.5)
IgG > N	1 (4.5)
Liver biopsy, n (%)	12 (54.5)
Time onset DILI - liver biopsy, days, mean (SD) (min-max)	44.1 (30.6) (8-126)
Hepatitis pattern, n (%)	
Hepatocellular	21 (95.5)
Mixed	1 (4.5)
Biology at the onset of hepatitis, mean (SD) (min-max)	
AST (IU/L)	341.3 (350) (34-1201)
ALT (IU/L)	562.7 (654.6) (59-2,523)
ALP (IU/L)	123.3 (58.5) (57-330)
GGT (IU/L)	89.5 (78.2) (17-336)
Total bilirubin (μmol/L)	11.7 (5.8) (4.5-25)
Biology at the peak of hepatitis, mean (SD) (min-max)	
AST (IU/L)	538.1 (346.7) (140-1443)
ALT (IU/L)	840.7 (605.5) (200-2,523)
ALP (IU/L)	154.9 (85.1) (80-423)
Corticosteroids n (%)	
No	13 (59.1)
Yes	9 (40.9)
Corticosteroids duration, days, mean (SD) (min-max)	60.0 (30.57) (15-103)
Improvement time, days, mean (SD) (min-max)	73.5 (54.2) (5-221)
CTCAE grade n (%)	
3/4	14 (63.6)/8 (36.4)
Severity (EWG grade), n (%)	
1 (mild)	18 (81.8)
2 (moderate)	3 (13.6)
3 (severe)	1 (4.6)
RUCAM score	6.64 (1.62)
Causality scale, n (%)	
Highly probable	7 (31.8)
Probable	10 (45.5)
Possible	5 (22.7)

ALP, alkaline phosphatase; ALT, alanine aminotransferase; AST, aspartate aminotransferase; DILI, drug-induced liver injury; GGT, gamma-glutamyltransferase.

significant clinical difference was observed between patients treated with abemaciclib and patients treated with ribociclib (Table S1). Patients also received concomitant endocrine therapy, mainly letrozole (n = 14), alone or in combination with triptorelin, leuprorelin or goserelin. Three patients were also taking herbal and dietary supplements (Chaga mushrooms and gemmotherapy, turmeric powder and unspecified phytotherapy); however, the causality assessment of these products did not make them a likely or a possible cause. The median time from the introduction of CDK4/6 inhibitors to hepatitis occurrence was 73 days (IQR 41.5–107.75) (Fig. S1). All patients underwent liver imaging (ultrasound, CT scan or MRI), and no biliary abnormalities or tumor progression (in the case of previously known liver metastases) were found. Viral serologies were negative in all patients. These parameters were either not present or not tested before initiation of anti-CDK4/6 therapy. In all cases, CDK4/6 inhibitors were permanently discontinued due to the liver injury.

Characteristics of hepatitis

Most of the patients were asymptomatic. Two patients were jaundiced and were admitted to hospital. All patients but one had a hepatocellular biological profile. Only one had a mixed pattern. The median peak was 717.5 IU/L (IQR 421.75–954.0) for ALT and 119.5 IU/L (IQR 106.25–157.25) for ALP.

Seven of 22 patients had autoimmune features: anti-nuclear antibodies (n = 5), anti-mitochondrial antibodies (n = 1) or increased IgG level (n = 2).

RUCAM scores were as follows: possible[3–5] in five (22.7%) cases, probable[6–8] in 10 (45.5%) cases and highly probable (>8) in seven (31.8%) cases. In one case, the RUCAM score was only 3 (possible) because pembrolizumab was used concomitantly, but CDK4/6 inhibitor-induced liver injury was considered probable.

Twelve (54.6%) patients had a liver biopsy within 44.1 ± 30.6 days from the onset of hepatitis. Liver biopsies were uninterpretable in two patients because of insufficient sampling. Other biopsies showed acute centrilobular hepatitis with foci of necrosis and lymphocytic infiltrate (Fig. 1). Biopsy characteristics are shown in Table 2.

According to the CTCAE classification, all patients exhibited grade 3 or 4 hepatitis. According to the DILI network severity classification, liver injury was classified as follows: mild in 18

A B

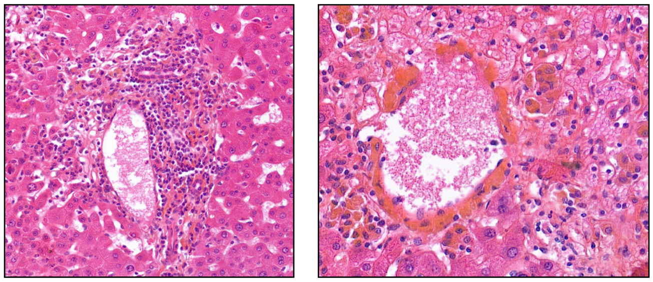

Fig. 1. Liver biopsy from patient with CD4/6-induced hepatitis. (A) Portal inflammation with periportal activity and hepatocellular loss. Inflammatory cells are represented by lymphocytes and a few plasma cells (HES x400). (B) Centrilobular confluent necrosis with detersion and hemorrhagic suffusions. Presence of lymphocytes at the interface with mediolobular hepatocytes (HES x400).

Table 2. Description of 10 liver biopsies; 2 were not interpretable.

Patient	Portal inflammation (0-3)	Duct injury (0/1)	Cholangitis 0/1)	Fibrosis (0/1)	Interface hepatitis (0-3)	Lobular hepatitis (0-3)	Confluent necrosis (0/1)	Infiltrate
**4**	1	rare	0	0	0	0	Peri centrolobular	
**5**	0	0	0	0	0	2	Rare	
**8**	0	0	0	0	0	1	Confluent necrosis	
**9**	1	0	0	0	0	0	0	Lymphocyte
**12**	1	0	0	0	0	0	0	Lymphocyte
**15**				Fibrosis with septa			Confluent necrosis	Lymphocyte
**16**	1	0	0	Peri sinusoidal	0	1	Peri centrolobular	Lymphocyte/eosinophils/plasmocyte
**17**	1	0	0	Fibrosis with septa	0	3	Rare	Eosinophils and neutrophils
**19**	2	0	0	Peri sinusoidal fibrosis	0	1	Confluent necrosis	Lymphocyte
**20**	2	0	1	Rare	0	0	Confluent necrosis	Neutrophils

patients (81.8%), moderate in three patients (13.6%) and severe in one patient (4.6%). There were no hepatitis-related deaths. In the case of the severe hepatitis leading to the hospitalization of the patient, the concomitant use of pembrolizumab and ribociclib may explain the severity of the disease. The recovery rate was 62.9% (95% CI 39.2-79.5) at day 45 and 39.2% (95% CI 17.8-60.2) at day 91, respectively (Fig. S2).

In five patients, anti-cancer therapy was resumed, including three patients treated with a CDK4/6 inhibitor (abemaciclib n = 2 and palbociclib n = 1), with follow-up between 72 and 212 days without hepatitis recurrence. When resuming treatment, a different CDK4/6 inhibitor was used from the one involved in hepatitis.

Effect of corticosteroid treatment

Nine (40.9%) patients were treated with corticosteroids, at a starting dose of 1 mg/kg in six patients and 0.5 mg/kg in three patients with a stepwise decrease to discontinuation. The decision to start steroids was made by the patient's oncologist or hepatologist, mainly in case of no improvement after discontinuation of the anti-CDK4/6 therapy. There was no difference in patients treated and non-treated with corticosteroids (Table 3). However, despite the limited number of patients, there was a trend for higher ALT levels (p = 0.08) and more liver biopsies (p = 0.09) in the corticosteroid-treated group. The median time from onset of liver injury to corticosteroid treatment initiation was 38 days (IQR 19–49). The median duration of steroid treatment was 70 days (IQR 36.0–77.5). The median time from the start of corticosteroid treatment to normalization of liver enzymes was 48 days (IQR 32.8–92.8) (Fig. 2). There was no significant difference in time to improvement between patients with autoimmune features compared to the others (83.1 (±61.1) *vs.* 52.7 (±29.2) days, p = 0.267). There was no recurrence of hepatitis when steroid administration was stopped, although one patient showed an increase in liver tests when steroids were reduced (secondary corticosteroid responder). Hepatitis improvement was significantly slower in patients treated with corticosteroids compared to non-treated patients (106.62 (±54.75) *vs.* 49.36 (±40.74); p = 0.009) (Table 3, Fig. 3). The progression of ALT elevations in all patients treated with corticosteroids is shown in Fig. 3. In the case of patient 7, who had a secondary increase in liver tests, corticosteroids were initially reduced more quickly.

Discussion

In the current study, we report the analysis of 22 cases of CDK4/6 inhibitor-induced liver injury. To date, only a few

Table 3. Characteristics of the patients according to corticosteroid treatment.

Characteristics	Anti-CDK4/6-induced liver injury without corticosteroid, n = 13	Anti-CDK4/6-induced liver injury with corticosteroid, n = 9	*p* value
Age, years, mean (SD)	61.5 (±14.9)	58.3 (±12.6)	0.48
Liver metastasis, n (%)			0.36
No	9 (69.2%)	8 (88.9%)	
Yes	4 (30.8%)	1 (11.1%)	
CDK4/6 inhibitor, n (%)			0.24
Abemaciclib	3 (23.1%)	0 (0.0%)	
Ribociclib	10 (76.9%)	9 (100.0%)	
Hormonal treatment, n (%)			0.92
Anastrozole	1 (7.7%)	1 (11.1%)	
Exemestane	0 (0.0%)	1 (11.1%)	
Fulvestrant	3 (23.1%)	1 (11.1%)	
Letrozole	4 (30.8%)	2 (22.2%)	
Letrozole/decapeptyl	2 (15.4%)	2 (22.2%)	
Letrozole/enantone	1 (7.7%)	2 (22.2%)	
Letrozole/gosereline	1 (7.7%)	0 (0.0%)	
Time CDK4/6 - DILI, days, mean (SD)	114.2 (±141)	91.0 (±45.8)	0.30
Autoimmune features, n (%)			0.27
No	10 (76.9%)	5 (55.6%)	
ANA ≥1/80	1 (7.7%)	4 (44.4%)	
ANA 1/160; AMA 1/320; IgG >N	1 (7.7%)	0 (0.0%)	
IgG >N	1 (7.7%)	0 (0.0%)	
Liver biopsy, n (%)			0.09
Yes	5 (38.5%)	7 (77.8%)	
No	8 (61.5%)	2 (22.2%)	
AST peak (IU/L), mean (SD)	508.9 (±352.0)	585.62 (±356.2)	0.37
ALT peak (IU/L), mean (SD)	672.4 (±488.7)	1,083.8 (±701.3)	0.08
ALP peak (IU/L), mean (SD)	174.2 (±104.5)	126.9 (±33.4)	0.48
Improvement time, days, mean (SD)	49.4 (±40.7)	106.6 (±54.8)	0.01
Severity (EWG grade), n (%)			0.74
1 (mild)	11 (84.6%)	7 (77.8%)	
2 (moderate)	2 (15.4%)	1 (11.1%)	
3 (severe)	0 (0.0 %)	1 (11.1%)	
CTCAE grade			0.66
3	9 (69.23%)	5 (55.56%)	
4	4 (30.77%)	4 (44.44%)	

ALP, alkaline phosphatase; ALT, alanine aminotransferase; AMA, anti-mitochondrial antibody; ANA, anti-nuclear antibody; AST, aspartate aminotransferase; DILI, drug-induced liver injury.

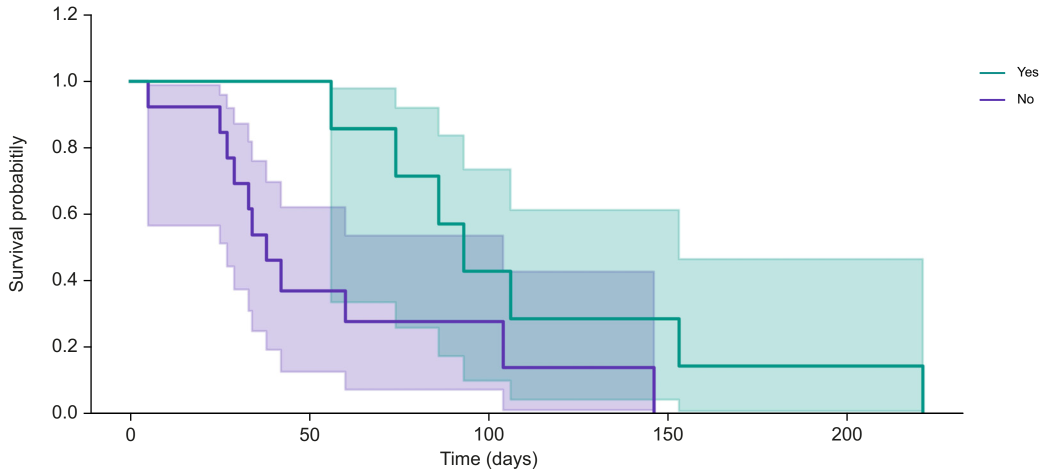

	No				
At risk	13	4	2	0	0
Censored	0	1	2	2	2
Events	0	8	9	11	11
	**Yes**				
At risk	9	8	3	2	1
Censored	0	1	2	2	2
Events	0	0	4	5	6

Fig. 2. Improvement of hepatitis after stopping CDK4/6 inhibitors according to steroid treatment.

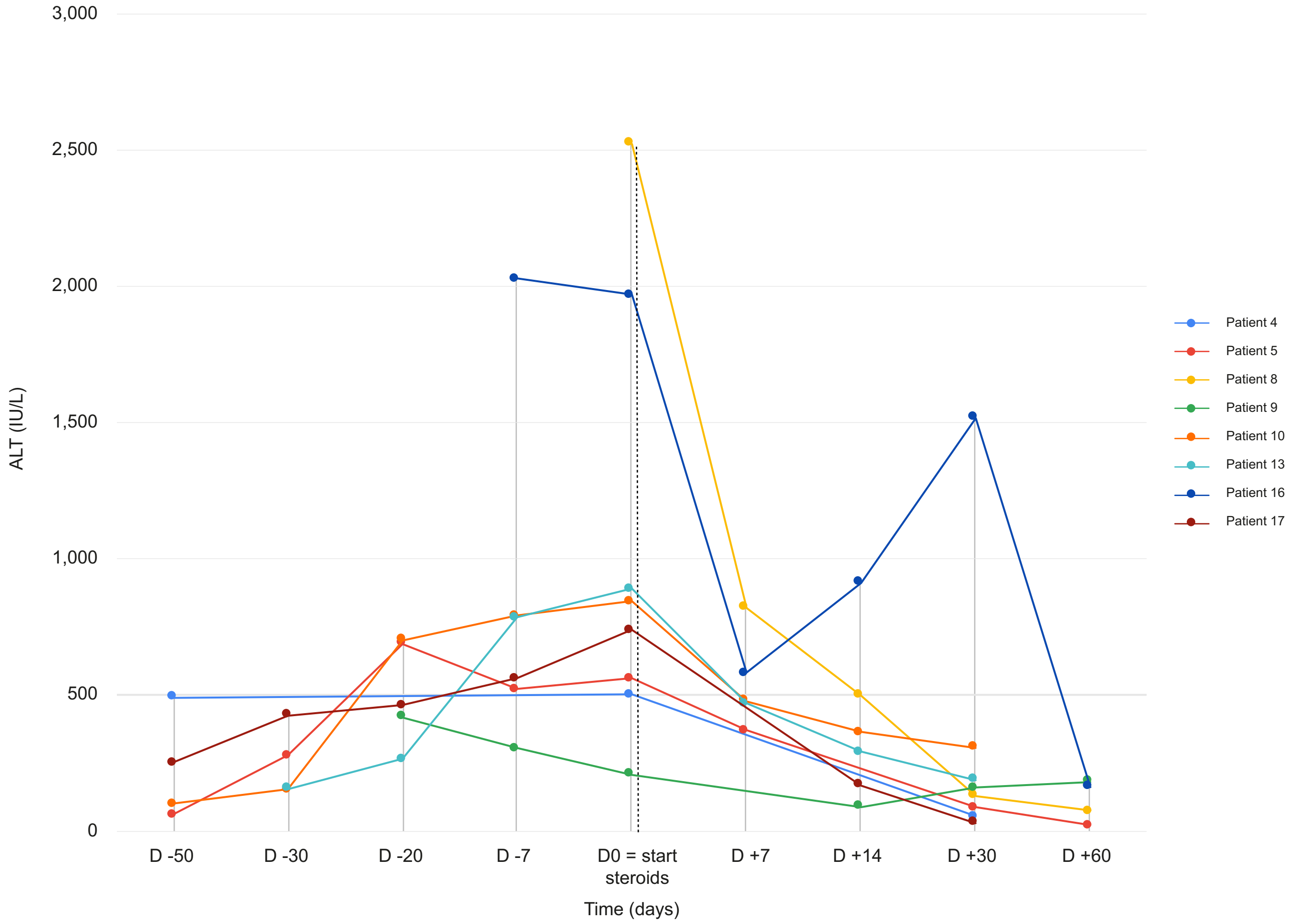

Fig. 3. Progression of ALT in patients treated with corticosteroids. Patient 15 is not shown because the precise dates of liver function tests after the introduction of corticosteroids are not available. ALT, alanine aminotransferase.

individual cases[11–13] have been published as well as a single series of six patients with rechallenge with another CDK4/6 inhibitor after hepatitis.[14]

Similarly to the available data in the literature, ribociclib followed by abemaciclib are the two CDK4/6 inhibitors mainly involved in our cohort. Palbociclib-induced liver injury has been reported in only one published case.[15]

In the present study, the predominant type of liver injury was hepatocellular and 32% of patients had autoimmune features, such as positive anti-nuclear antibodies and/or elevated serum IgG levels at the time of diagnosis.

A corticosteroid treatment was initiated in 40% of patients, independently of the presence of autoimmune features. Finnsdottir S *et al.*, described for the first time in 2021 the benefits of corticosteroids in the treatment of ribociclib-induced hepatitis.[11] In our study, patients treated with corticosteroids had a slower improvement than untreated patients. Corticosteroids allowed for the resolution of liver injury that had not improved spontaneously.

Recently, the International Autoimmune Hepatitis Group (IAIHG) and the Drug and Herbal & Dietary Supplement-induced Liver Injury consortium (EASL DHILI) have developed recommendations for the diagnosis, description and management of drug-induced autoimmune-like hepatitis (DI-ALH).[16] CDK4/6 inhibitors are not listed in these recommendations but there are several arguments that could point out that these drugs should be included in the DI-ALH category. Indeed, evidence of autoimmunity was found in one-third of patients, although not all were tested for it. Histopathological findings revealed by liver biopsy and biological phenotype of liver injury are also consistent with this mechanism. The response to corticosteroids and the absence of recurrence of hepatitis after corticosteroid discontinuation is an additional argument. A parallel can be established with infliximab-induced hepatitis, where corticosteroids are also administered in the absence of spontaneous recovery, whatever the autoimmune background.[17]

As already reported in the literature,[12,14,18] there seems to be no class effect in anti-CDK4/6 rechallenge after hepatitis. In our series, three patients resumed an CDK4/6 inhibitor after resolution of liver injury, using another CDK4/6 inhibitor (palbociclib or abemaciclib) without relapse. In this series, no patient experienced fulminant hepatitis or death secondary to anti-CDK4/6-induced liver damage. According to our results, regular and extended monitoring of liver tests should be carried out in patients treated with anti-CDK4/6. In case of hepatitis grade 3 or 4, treatment should be discontinued. Corticosteroids should be discussed in the absence of spontaneous improvement. In our series, corticosteroids were started about 1 month after the onset of hepatitis. We acknowledge some limitations to these results: the number of patients is limited, and management is heterogeneous, particularly as regards the indication for liver biopsy and corticosteroids.

In conclusion, CDK4/6 inhibitors are mostly responsible for asymptomatic hepatocellular liver injury, associated with the presence of autoantibodies in up to one-third of cases.

Our findings suggest that in cases where there is no improvement in liver injury following the withdrawal of CDK4/6 inhibitors, corticosteroid therapy may potentially aid in the recovery process, although further studies are needed to confirm this.

Furthermore, in patients with a history of anti-CDK4/6-induced liver injury, exposure to another CDK4/6 inhibitor does not seem to be associated with hepatitis relapse, suggesting the absence of cross-direct hepatotoxicity or the development of an adaptation to this drug family. Nevertheless, we suggest frequent monitoring of liver function tests in case of resumption of another CDK4/6 after hepatitis.

Affiliations

[1]*Liver Unit, Saint-Eloi Hospital, INSERM 1183, Montpellier School of Medicine, Montpellier, France;* [2]*AP-HP Hôpital Paul-Brousse, Centre Hépato-Biliaire, INSERM Unit 1193, Université Paris-Saclay, FHU Hepatinov, Villejuif, F-94800, France;* [3]*Department of Gastroenterology, Cliniques Universitaires Saint-Luc et Institut de Recherche Clinique (IREC), Université Catholique de Louvain, Brussels, Belgium;* [4]*Department of Medical Oncology, Montpellier Cancer Institute Val d'Aurelle, INSERM U1194, Montpellier University, 208 rue des Apothicaires, F-34298 Montpellier, France*

Abbreviations

ALP, alkaline phosphatase; ALT, alanine aminotransferase; AST, aspartate aminotransferase; DILI, drug-induced liver injury; CDK4/6, cyclin-dependent kinase 4/6; HR+, hormone receptor-positive; HER2-, human epidermal growth factor receptor-2-negative; RUCAM, Roussel-Uclaf causality assessment method; ULN, upper limit of normal.

Financial support

AFEF (Association Française pour l'Etude des maladies du Foie) funding.

Conflicts of interest

The authors declare no conflicts of interest related to this work.

Please refer to the accompanying ICMJE disclosure forms for further details.

Authors' contributions

LM, EDM, BD, YH and DL: study conception and design. LM, BD, AZ, SG, WJ and EDM: data acquisition. LM, EDM, BD, YH and DL: data analysis and interpretation. LM, EDM, BD, YH and DL: manuscript preparation and drafting. AZ, LM: statistical data analysis. All authors: manuscript reviewing. All authors have approved the final manuscript submitted.

Data availability statement

The data that support the findings of this study are available on request from the corresponding author.

Ackowledgements

All the doctors who have contributed to REFHEPS: Angélique Bobrie, Marie Alexandre, Marie Viala, Véronique D'hondt, Sandrine Barge, Philine Witkowski, Yanis Dahel, and Sarah Cavaillon. Catherine Guettier (liver biopsy). Patients and their families.

Supplementary data

Supplementary data to this article can be found online at https://doi.org/10.1016/j.jhepr.2024.101098.

References

[1] Hortobagyi GN, Stemmer SM, Burris HA, et al. Ribociclib as first-line therapy for HR-positive, advanced breast cancer. N Engl J Med 2016 Nov 3;375(18):1738–1748.

[2] Goetz MP, Toi M, Campone M, et al. Monarch 3: abemaciclib as initial therapy for advanced breast cancer. J Clin Oncol Off J Am Soc Clin Oncol 2017 Nov 10;35(32):3638–3646.

[3] Rugo HS, Huober J, García-Sáenz JA, et al. Management of abemaciclib-associated adverse events in patients with hormone receptor-positive, human epidermal growth factor receptor 2-negative advanced breast cancer: safety analysis of MONARCH 2 and MONARCH 3. The Oncologist 2021 Jan;26(1):e53–e65.
[4] Verma S, Bartlett CH, Schnell P, et al. Palbociclib in combination with fulvestrant in women with hormone receptor-positive/HER2-negative advanced metastatic breast cancer: detailed safety analysis from a multicenter, randomized, placebo-controlled, phase III study (PALOMA-3). The Oncologist 2016 Oct;21(10):1165–1175.
[5] Cristofanilli M, Turner NC, Bondarenko I, et al. Fulvestrant plus palbociclib versus fulvestrant plus placebo for treatment of hormone-receptor-positive, HER2-negative metastatic breast cancer that progressed on previous endocrine therapy (PALOMA-3): final analysis of the multicentre, double-blind, phase 3 randomised controlled trial. Lancet Oncol 2016 Apr;17(4):425–439.
[6] Andrade RJ, Aithal GP, Björnsson ES, et al. EASL clinical practice guidelines: drug-induced liver injury. J Hepatol 2019 Mar 26;70(6):1222–1261.
[7] Hoofnagle JH, Björnsson ES. Drug-induced liver injury - types and phenotypes. N Engl J Med 2019 Jul 18;381(3):264–273.
[8] Danan G, Teschke R. RUCAM in drug and herb induced liver injury: the update. Int J Mol Sci 2015 Dec 24;17(1).
[9] Fontana RJ, Seeff LB, Andrade RJ, et al. Standardization of nomenclature and causality assessment in drug-induced liver injury: summary of a clinical research workshop. Hepatol Baltim Md 2010 Aug;52(2):730–742.
[10] Common terminology criteria for adverse events (CTCAE) version 5.0. https://ctep.cancer.gov/protocolDevelopment/electronic_applications/docs/CTCAE_v5_Quick_Reference_5x7.pdf.
[11] Finnsdottir S, Sverrisdottir A, Björnsson ES. Hepatotoxicity associated with ribociclib among breast cancer patients. Acta Oncol Stockh Swed 2021 Feb;60(2):195–198.
[12] Farhat F, Tarabaih M, Kanj A, et al. Palbociclib safety and efficacy beyond Ribociclib-induced liver toxicity in metastatic hormone-receptors positive breast cancer patient. Anticancer Drugs 2020 Jan;31(1):85–89.
[13] Er MM, Araz M, Hendem E, et al. Ribociclib-induced hepatotoxicity. J Oncol Pharm Pract Off Publ Int Soc Oncol Pharm Pract 2023 Jul;29(5):1275–1277.
[14] Fuentes-Antrás J, de Luna A, López de Sá A, et al. Safety and efficacy of cyclin-dependent kinase inhibitor rechallenge following ribociclib-induced limiting hypertransaminasemia. Breast Edinb Scotl 2020 Dec;54:160–163.
[15] Hyppolite JJ, Hilzenrat N. Palbociclib-induced severe hepatitis: a case study and literature review. Can Liver J 2021;4(4):433–437.
[16] Andrade RJ, Aithal GP, Boer YS de, et al. Nomenclature, diagnosis and management of drug-induced autoimmune-like hepatitis (DI-ALH): an expert opinion meeting report. J Hepatol 2023 Sep 1;79(3):853–866.
[17] Björnsson HK, Gudbjornsson B, Björnsson ES. Infliximab-induced liver injury: clinical phenotypes, autoimmunity and the role of corticosteroid treatment. J Hepatol 2022 Jan;76(1):86–92.
[18] Meynard L, Grellety T. CDK 4/6 inhibitor successful rechallenge after limiting hepatic toxicity. Breast J 2020 Feb;26(2):255–257.

Keywords: DILI; breast cancer; hepatotoxicity; Cyclin-dependent kinase inhibitors; corticosteroids.
Received 25 November 2023; received in revised form 2 April 2024; accepted 5 April 2024; Available online 16 April 2024

Journal of Hepatology, Volume *6*

Supplemental information

CDK4/6 inhibitor-induced liver injury: Clinical phenotypes and role of corticosteroid treatment

Lucy Meunier, Eleonora De Martin, Bénédicte Delire, Wiliam Jacot, Severine Guiu, Amel Zahhaf, Dominique Larrey, and Yves Horsmans

CDK4/6 inhibitor-induced liver injury: Clinical phenotypes and role of corticosteroid treatment

Lucy Meunier, Eleonora De Martin, Bénédicte Delire, Wiliam Jacot, Severine Guiu, Amel Zahhaf, Dominique Larrey, Yves Horsmans

Table of contents

Fig. S1……………………………………………………………………………………2

Fig. S2……………………………………………………………………………………2

Fig. S1. Onset of hepatitis after CDK4/6 inhibitors

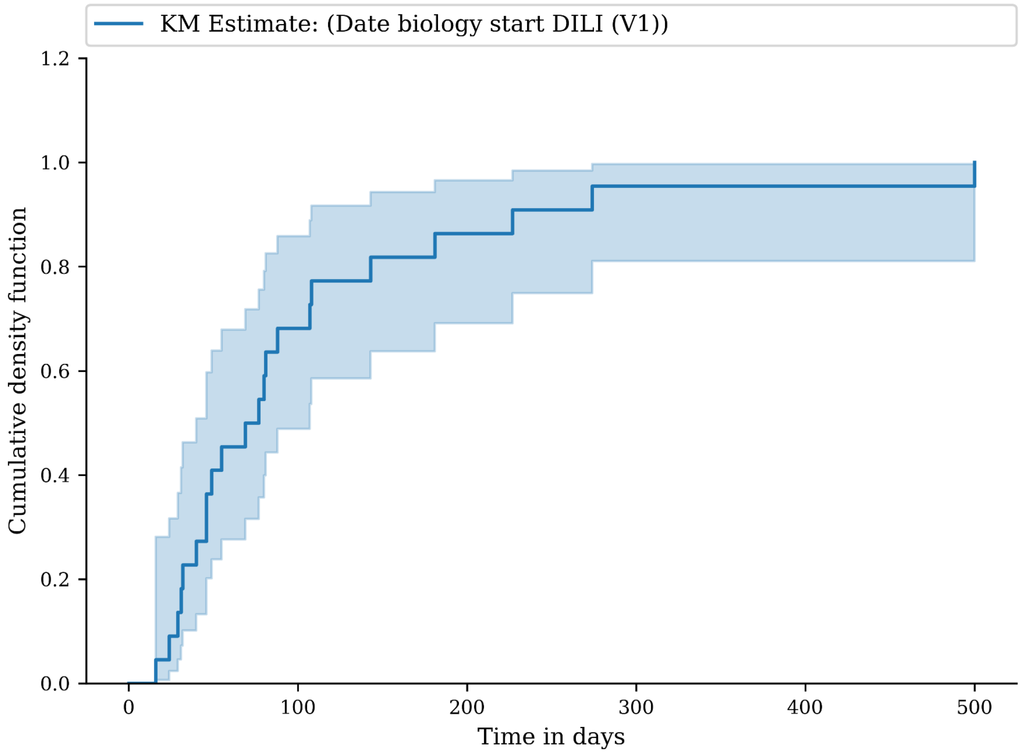

Fig. S2. Improvement of hepatitis after stopping CDK4/6 inhibitors

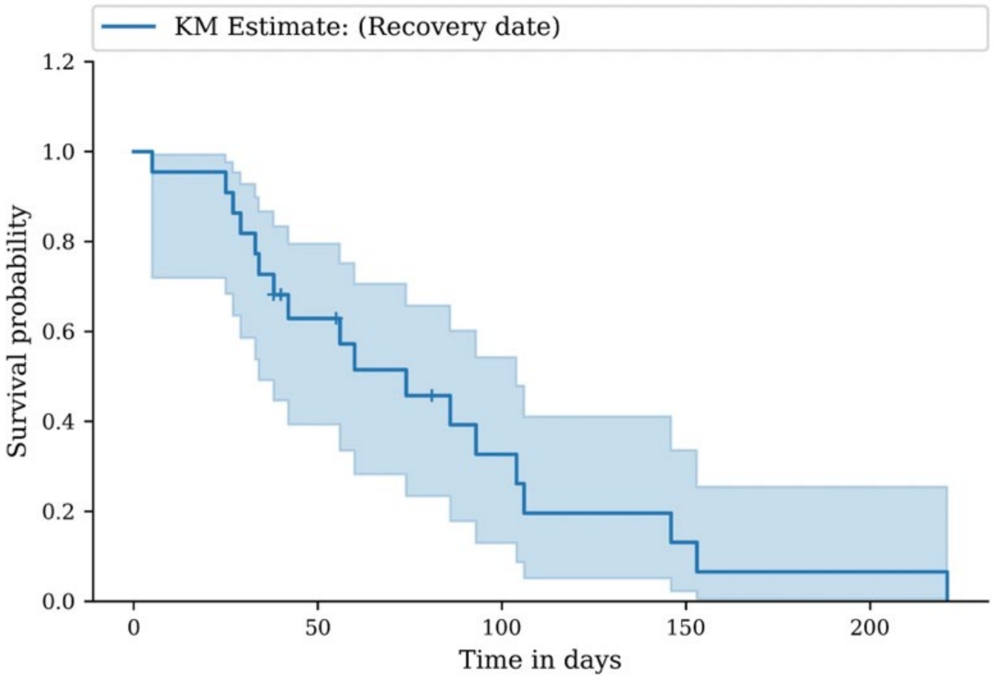

KM Estimate:

At risk	22	12	5	2	1
Censored	0	2	4	4	4
Events	0	8	13	16	17
